# Predictors of Dietary Supplement Usage among Medical Interns of Tehran University of Medical Sciences

**Published:** 2015-03

**Authors:** Gity Sotoudeh, Sanaz Kabiri, Haleh Sadrzadeh Yeganeh, Fariba Koohdani, Farahnaz Khajehnasiri, Shahla Khosravi

**Affiliations:** ^1^Department of Community Nutrition, School of Nutritional Sciences and Dietetics, Tehran University of Medical Sciences, Tehran, Iran; ^2^Department of Social Medicine, School of Medicine, Tehran University of Medical Sciences, Tehran, Iran; ^3^Department of Cellular, Molecular Nutrition, School of Nutritional Sciences and Dietetics, Tehran University of Medical Sciences, Tehran, Iran

**Keywords:** Dietary supplement, Intern, Lifestyle, Tehran University of Medical Sciences, Iran

## Abstract

This study aimed to determine the prevalence of dietary supplement-use and its relationship with demographics and lifestyle of medical interns. The study sample comprised 356 interns aged 23 to 25 years. Participants completed a questionnaire on dietary supplement-use during the month preceding the study, information on demographic characteristics and lifestyle was also obtained. Univariable and multivariable logistic regression were employed to assess the correlates of dietary supplement-use. The prevalence of dietary supplement-use was about 33% (males 20.4% and females 43.2%, p<0.001). The most commonly-used dietary supplement was multivitamin/multivitamin-mineral (90.6% in males and 52.3% in females). Approximately 30% of supplements were used regularly (≥5 days/week) by all subjects. The most-frequently reported reasons for supplement-use in males were: enhancing daily energy/stamina (51.1%), poor food intake (13.3%) and, in females, were: improving health and nutritional status (39.3%) and reducing hair loss (23.4%). The decision to use dietary supplement was mostly driven by the interns themselves (56% in males, 61% in females). In the univariable analysis, men who exercised once or twice a week were less likely to use supplements compared to those who reported doing exercise more than twice weekly (OR=0.35, 95% CI 0.12-0.98). Females who reported their health status to be ‘excellent’ were more likely to use supplements compared to those who described their health status as ‘moderate/poor/very poor’ (OR=2.53, 95% CI 1.15-5.56) as were women who mentioned their breakfast consumption status as ‘always’ (OR=2.69, 95% CI 1.47-4.92). In the multivariable analysis, only breakfast consumption was significantly related with dietary supplement-use in females (OR=2.20, 95% CI 1.11-4.38). In conclusion, dietary supplement-use among medical interns, especially among females, was relatively very common. Dietary supplement-use was related to a healthier lifestyle.

## INTRODUCTION

According to the 1994 US Dietary Supplement Health and Education Act, a nutritional supplement is defined as a product intended to supplement the diet, containing one or more dietary ingredient(s), including vitamins, minerals, herbs, amino acids, or other botanicals, and to be taken by mouth as a pill, capsule, tablet, or liquid (1). Dietary supplement-use is prevalent worldwide (2,3). About 50% of Americans consume multivitamin supplements at least weekly (4). Some reported reasons for taking dietary supplement include providing nutritional needs (5), preventing disease (3,6), increasing energy/stamina, ensuring good health (5), and enhancing athletic performance (7,8).

Although some people may benefit from taking dietary supplements in certain periods of their lives, the use of these supplements by others may have no beneficial effects. Excessive use of dietary supplements may lead to adverse effects, such as increasing risk of cancer (9–12), liver disease (13), and hypervitaminosis (14), among other conditions.

In recent years, a wide variety of supplements has been available in Iran, and taking these supplements seems to be gaining popularity. However, a few studies are available on the prevalence of these supplement-use and the characteristics of the supplement-users. The results of some studies in Iran indicated that the prevalence of dietary supplement-use among special groups were 45-77% (15–17).

Medical interns usually spend a lot of time for hospital work in their internship period, and many of them live in dormitory in which the quality of the provided food is poor. Therefore, they may tend to consume more dietary supplements which are important for promoting their health. Furthermore, their health practices may affect the health of their future patients. It is worthy to understand the patterns of dietary supplement-use in various populations to better understand the types and frequency of supplement-use, and their potential adverse effects differ among populations. This study was carried out to assess the dietary supplement intake by medical interns of Tehran University of Medical Sciences, Iran. The correlations of supplement-use such as demographic and lifestyle characteristics, were also studied.

## MATERIALS AND METHODS

This study was conducted from June to July 2009 at Tehran University of Medical Sciences, one of the main state universities located at the centre of Tehran. All 400 interns aged 23 to 25 years from the School of Medicine were invited to participate. The study was approved by the Medical School's Deputy Chancellor for Research. Interns completed a self-administered questionnaire in which they were asked about the types of dietary supplements they ingested. The participants were provided with a definition of what are considered dietary supplements which included vitamins, minerals, protein, omega-3, and fibre. We excluded consideration of other dietary supplement intake, such as energy drink, herbs, etc. Supplement-use was defined as taking ≥1 times/week of any mentioned supplements during the previous month.

The subjects were asked about the reasons for taking the supplements, the frequency of taking the supplements per week, and about the person(s) influencing their decision to take the supplements. Data on demographic factors and lifestyle were collected. These included: marital status (single, married, and widowed or divorced), residence status (home or dormitory), period of internship (less than 6, 6-12, and 13-18 months), and current smoking habits, including cigar, hookah, and pipe tobacco (yes or no). In addition, subjects were asked about their habits of breakfast consumption; response categories were: always, occasionally, and never. For statistical analysis, the responses were collapsed into 2 categories: always and occasionally versus never. The individual's perception of health status was determined by asking “how would you rate your overall health status: excellent, good, average, poor, or very poor?” The participants’ responses were then categorized in three levels of excellent, good, moderate/poor/very poor. Physical activity was measured by asking a question about the level of physical activity; their responses were then categorized as 1=no exercise, 2=occasional exercise; hobbies or sports requiring exercise once or twice a week, 3=regular exercise 3 times a week or more. The final samples consisted of 356 interns with an 89% response rate.

### Statistical analysis

Statistical analyses were carried out using SPSS 11.5 for Windows (Chicago, IL, USA). Crude odds ratios with 95% confidence intervals were calculated in order to analyze associations among dietary supplement-use and demographic and lifestyle variables. Multivariable analyses were conducted using a logistic regression model to assess the correlates of supplement-use in males and females. Differences were considered significant at p<0.05.

## RESULTS

Supplement-use was significantly higher in women than in men (43.2% vs 20.4%, p<0.001). The most commonly-used dietary supplement was multivitamin/multivitamin-mineral (90.6% in males and 52.3% in females), followed by iron and zinc in females (43 and 34.9% respectively). In addition, 18.8% of males and 54.6% of females reported the intake of more than one supplement in the preceding month. Supplements of other nutrients, such as vitamin E, magnesium, selenium, and protein, were used infrequently (Table 1).

Approximately 30% of supplements were used regularly (≥5 days/week) by all subjects. The most commonly-reported reasons for supplement-use in males were: enhancing daily energy/stamina (51.1%), poor food intake (13.3%). Females mentioned improving health and nutritional status (39.3%) and loss of hair (23.4%) as the most common reasons for taking supplements. The decision to use dietary supplement was mostly driven by the interns themselves (56% in males, 61% in females) (Table 2).

Men who reported physical activity once or twice a week were less likely to use supplements compared to those who reported physical activity more than twice a week (OR=0.35, 95% CI 0.12-0.98) (Table 3). Females who reported their health status to be ‘excellent’ were more likely to use supplements compared to those who described their health status as ‘moderate/poor/very poor’ (OR=2.53, 95% CI 1.15-5.56) as were women who mentioned their breakfast consumption status as ‘always’ (OR=2.69, 95% CI 1.47-4.92). In multivariable analysis, breakfast consumption was significantly related with dietary supplement-use in females (OR=2.20, 95% CI 1.11-4.38). In men, the association between physical activity and supplement-use had borderline statistical significance (Table 4).

**Table 1.** Prevalence of dietary supplement-use in the month preceding the study among interns of Tehran University of Medical Sciences, 2009

Supplement type	Supplement-use	
	Males (N=32)	Females (N=86)
	No. (%)	No. (%)
Multivitamin/Multivitamin-multiminerals	29 (90.6)	45 (52.3)
One of B vitamins/B complex vitamins	1 (3.1)	15 (17.4)
Vitamin D/Vitamin D + Calcium	1 (3.1)	7 (8.1)
Calcium	0 (0.0)	8 (9.3)
Iron	1 (3.1)	37 (43.0)
Zinc	4 (12.5)	30 (34.9)
Omega-3	2 (6.2)	6 (7.0)
Magnesium/Selenium/Vitamin E/Protein	0 (0.0)	4 (4.6)
More than one supplement intake	6 (18.8)	47 (54.6)
Total N=356		

**Table 2.** Reasons of dietary supplement-use and the person(s) influencing intern's decision to take the supplements in the month preceding the study among interns of Tehran University of Medical Sciences, 2009

Reason	Supplement-use	
	Males (N=32)	Females (N=86)
	No. (%)	No. (%)
Desire to lose weight	3 (6.7)	6 (4.1)
Desire to gain weight	2 (4.4)	0
Enhancing daily energy/stamina	23 (51.1)	12 (8.3)
Improving athletic performance	2 (4.4)	2 (1.4)
Improving health and nutritional status	2 (4.4)	57 (39.3)
Disease treatment	5 (11.2)	14 (9.6)
Preventing hair loss	2 (4.4)	34 (23.4)
Poor food intake	6 (13.4)	13 (9.1)
Improving skin health	0 (0.0)	7 (4.8)
Person(s) influencing intern's decision		
Physician	6 (13.3)	43 (29.6)
Nutritionist/Dietitian	7 (15.6)	13 (9)
Family/Friends	2 (4.4)	11 (7.6)
Self	25 (55.6)	88 (60.7)
Total N=356		

**Table 3.** Association between dietary supplement-use and demographic and lifestyle characteristics in male interns, 2009

Variable	Supplement-use (N=32) No. (%)	Unadjusted OR* (95% CI)†	p value	Adjusted OR‡ (95% CI)	p value
Marital status					
Single	16 (16.5)	0.54 (0.24-1.19)	0.1	0.50 (0.21-1.17)	0.1
Married	16 (26.7)	1		1	
Residence status					
Home	24 (21.6)	1.31 (0.54-3.17)	0.5	1.13 (0.44-2.87)	0.7
Dormitory	8 (17.4)	1		1	
Period of internship (months)					
<6	7 (19.4)	1.25 (0.43-3.56)	0.6	1.42 (0.47-4.28)	0.5
6-12	14 (26.4)	1.86 (0.76-4.52)	0.1	1.72 (0.67-4.36)	0.2
13-18	11 (16.2)	1		1	
Smoking					
Yes	18 (23.7)	1.48 (0.68-3.20)	0.3	1.61 (0.69-3.72)	0.2
No	14 (17.3)	1		1	
Physical activity (times/week)					
0	8 (17.4)	0.34 (0.10-1.09)	0.07	0.30 (0.08-1.07)	0.06
1-2	16 (17.8)	0.35 (0.12-0.98)	0.04	0.39 (0.13-1.20)	0.1
3 and more	8 (38.1)	1		1	
Perceived health status					
Excellent	4 (15.4)	0.63 (1.17-2.28)	0.4	0.49 (0.12-1.98)	0.3
Good	18 (20.9)	0.92 (0.38-2.22)	0.8	0.78 (0.30-1.99)	0.6
Moderate/Poor/Very poor	10 (22.2)	1		1	
Breakfast consumption					
Always	16 (20.8)	1.04 (0.48-2.28)	0.9	0.87 (0.37-2.04)	0.7
Occasionally/Never	16 (20.0)	1		1	
Total N=356					

*Odds ratio

†Confidence interval

‡Adjusted for marital status, residence, period of internship, smoking, physical activity, perceived health status, and breakfast consumption

## DISCUSSION

To our knowledge, this study is the first to document dietary supplement-use in medical interns in Iran. The results of this study indicated that about 33% of interns used dietary supplements in the month preceding the study. Compared to males, females had more than twice dietary supplement-use, and about half of them reported more than one supplement intake. Multivitamins/multivitamins-minerals were the most commonlyused dietary supplements in both sexes. Multivariable analyses revealed that breakfast consumption was the only determinant of supplement-use in women. In men, those who exercised less than 3 times a week were less likely to use dietary supplements; however, the difference was of borderline statistical significance. Gender differences in dietary supplement-use have been described before. For example, higher prevalence of supplement-use in women compared to men has been found in fitness club participants in Iran (16), Malaysian, American and Jordanian students (18–20), and South Korean and American adults (21–23). However, vitamin supplement-use was higher in men than women in Portuguese students of health science (24). It is possible that women use dietary supplements more often than men since they may be more concerned about their nutrients intake. Gender differences in lifestyle have been shown in other studies (25–27). Female students in Canada had higher consumption breakfast and lower soft drink and energy drink compared to male students (25). Swedish female students showed healthier habits of alcohol consumption and nutrition but they were more stressed. Male students had unhealthy nutritional habits and less physical activity level (26). Greek female students exercised less than males but they had healthier eating habits and lower alcohol consumption (27).

**Table 4.** Association between dietary supplement-use and demographic and lifestyle characteristics in female interns, 2009

Variable	Supplement-use (N=86) No. (%)	Unadjusted OR* (95% CI)†	p value	Adjusted OR‡ (95% CI)	p value
Marital status					
Single	63 (43.8)	1.08 (0.57-2.02)	0.8	0.95 (0.48-1.91)	0.9
Married	23 (41.8)	1		1	
Residence status					
Home	68 (47.2)	1.83 (0.95-3.52)	0.06	1.61 (0.80-3.21)	0.1
Dormitory	18 (32.7)	1		1	
Period of internship (months)					
<6	26 (45.6)	1.17 (0.59-2.31)	0.6	1.08 (0.51-2.30)	0.8
6-12	25 (43.1)	1.06 (0.53-2.08)	0.8	0.98 (0.48-2.01)	0.9
13-18	35 (41.7)	1		1	
Smoking					
Yes	13 (40.6)	1.13 (0.52-2.44)	0.7	1.30 (0.57-2.97)	0.5
No	73 (43.7)	1		1	
Physical activity (times/week)					
0	27 (47.4)	0.84 (0.34-2.05)	0.7	0.90 (0.34-2.35)	0.8
1-2	44 (38.9)	0.59 (0.26-1.35)	0.2	0.57 (0.24-1.38)	0.2
3 and more	15 (51.7)	1		1	
Perceived health status					
Excellent	26 (55.3)	2.53 (1.15-5.56)	0.02	1.82 (0.74-4.44)	0.1
Good	40 (44.0)	1.60 (0.81-3.16)	0.1	1.34 (0.65-2.78)	0.4
Moderate/Poor/Very poor	20 (32.8)	1		1	
Breakfast consumption					
Always	63 (52.5)	2.69 (1.47-4.92)	0.001	2.20 (1.11-4.38)	0.02
Occasionally/Never	23 (29.1)	1		1	
Total N=356					

*Odds ratio

†Confidence interval

‡Adjusted for marital status, residence, period of internship, smoking, physical activity, perceived health status, and breakfast consumption

It is for the first time that the current study revealed the positive relationship between regular breakfast intake as a healthy habit in women and supplement-use. Except this behaviour, our results are not similar to those from other studies where dietary supplement-users have a healthier lifestyle, such as lower smoking habit and higher physical activity level (20,23–24,28–30).

The study in Portuguese students also showed no lifestyle difference between users and non-users of supplements (24).

Iron supplements were consumed by 43% of women, which is lower than Iranian female fitness club participants (16) and much higher than female physicians in the United States (17%) (31). This higher percentage of iron supplement intake may reflect more prevalence of iron deficiency in female students. However, only less than 10% of females mentioned the reason of ‘disease treatment’ for their dietary supplement-use.

Findings of the present study in females are consistent with the results of other studies since it reports improving health and nutritional status as the most common reason for dietary supplement-use (5,16). The most commonly-reported reason for taking supplements was: enhancing daily energy/stamina for men. This may be due to their higher physical activity levels. Male fitness club participants in Iran also reported increasing energy as a reason for their dietary supplement-use (16).

In univariable analysis, the use of supplements in women was not associated with physical activity level whereas, in men, physical activity level was higher among users. Other studies found higher use of dietary supplement among individuals who were more physically active (20,22,23,28,29). However, more investigation on physical activity level and supplement-use is required. Furthermore, women with perceived better health status and regular intake of breakfast had a greater likelihood of reporting the use of dietary supplements. Consistent with other studies (23,32,33), the findings of the present study showed better perception of health status among supplement-users in univariable analysis. However, the relationship between supplement intake and chronic diseases has been shown in one of these studies (33).

The frequency of supplement-use in our study is lower than the frequencies among Iranian female health workers and collegiate athletes (17), pharmacy and medicine students, and physicians in the United States (19,28,31,34), adults in Alberta (35) and Korea (21) and higher than those in Jordan (20), Portuguese students of health science (24), and Malaysian students of physical education (18).

Multivitamins/multivitamins-minerals were the most frequently-used dietary supplements by participants. This finding is consistent with the data reported from Iran (16,17), the United States (5,23), and Jordan (20). This supplement, as the main reported supplement used among interns, indicates that they may be concerned about their nutritional adequacy in general. Thus, they selected a type of supplement providing a variety of nutrients. Among female health workers, iron was the most frequently-used supplement, and ‘disease treatment, and health/nutritional status promotion’ was the most reported reasons for supplement intake (15).

Most supplements were purchased by the interns themselves. As interns were exposed to clinical studies that showed the beneficial effects of dietary supplements on the prevention and treatment of diseases, they may have considered themselves to be candidates for potential benefits of supplement-use. In addition, they might consider themselves as physicians who are eligible for prescribing dietary supplements without any supervision.

Supplement-use was not related with the period of internship. As the period of internship increased, higher prevalence of supplement-use was expected. Longer internship period may imply greater awareness of the role of nutrition in wellbeing. However, a study which was designed to evaluate resident physicians’ level of understanding of the efficacy, safety, and drug-supplement interactions in common dietary supplement showed poor knowledge in medical residents in the United States (average score 59.7%) (36). Since healthcare providers, including physicians, were reported as the most-cited factor influencing participants’ decision to take dietary supplements (16,19,20), their knowledge and practices in this issue are very important.

The prevalence of smoking was not different between users and non-users of supplements. Other studies suggest a negative relationship between dietary supplement-use and smoking habit (20,22,29,30,33). However, Portuguese male students who were supplement-users had higher smoking habits (24).

Contrary to our expectation, supplement-use in interns living at dormitories was not different from those living at home. The types of food which were available to interns at dormitory were of poor quality. Dietary supplement, if taken properly, could be beneficial when diet does not meet nutritional requirements for disease prevention and treatment. It was, therefore, expected that interns who lived at dormitories would have higher supplement-use. However, this finding should be investigated in more details.

There was no relationship between marital status and supplement-use but, in a study of elderly Japanese, the results revealed that married men were more likely to take vitamins compared to singles (37). In another study, unmarried subjects were more likely to use herbs than those who were married but this difference was not seen in vitamin or mineral supplement-use (38).

### Strengths and limitations

This study had some limitations. First, the study was conducted on interns from only one university, which may not be the representative of all Iranian interns. Second, physical activity was measured briefly, which may not reflect actual physical activity level of the subjects. Third, data on supplement-use were self-reported, which can result in under- or overreporting of supplement-use. Fourth, we did not ask the subjects about the presence of any chronic illnesses, and we know it may affect the results. Lastly, given the cross-sectional study design, inference in causality is limited. Despite these limitations, this study provides some data on dietary supplement-use in a special group of healthcare providers, which may have some effects on their patients’ health as well.

### Conclusions

The use of dietary supplements among medical interns was relatively high. Only breakfast consumption was significantly related with dietary supplement-use in females. The results of this study imply that medical interns may need more education on prescription-indication and safety of dietary supplements. More detailed studies are recommended for assessment of dietary supplement-use and its relationship with lifestyle, including dietary patterns in medical interns.

## ACKNOWLEDGEMENTS

This study was funded and supported by School of Medicine, Tehran University of Medical Sciences.

**Conflict of interest:** Authors have no conflicts of interest.

## References

[1] Dietary Supplement Health and Education Act of 1994 (1994). Public Law 103-417.

[2] Fraunfelder FW (2005). Ocular side effects associated with dietary supplements and herbal medicines. Drugs Today (Barc).

[3] Greenwald P, Anderson D, Nelson SA, Taylor PR (2007). Clinical trials of vitamin and mineral supplements for cancer prevention. Am J Clin Nutr.

[4] Murphy SP, White KK, Park SY, Sharma S (2007). Multivitamin-multimineral supplements’ effect on total nutrient intake. Am J Clin Nutr.

[5] Tsang SN, Pycz LA, Herbold NH (2007). Dietary supplement use among physically active multiethnic adults. Topics Clin Nutr.

[6] Prentice RL (2007). Clinical trials and observational studies to assess the chronic disease benefits and risks of multivitamin-multimineral supplements. Am J Clin Nutr.

[7] Position of the American Dietetic Association, Dietitians of Canada, and the American College of Sports Medicine: nutrition and athletic performance (2000). J Am Diet Assoc.

[8] Dorsch KD, Bell A (2005). Dietary supplement use in adolescents. Curr Opin Pediatr.

[9] Thompson J, Manore M (2005). Nutrition: an applied approach.

[10] Mulholland CA, Benford DJ (2007). What is known about the safety of multivitamin-multimineral supplements for the generally healthy population? Theoretical basis for harm. Am J Clin Nutr.

[11] (1994). The effect of vitamin E and beta carotene on the incidence of lung cancer and other cancers in male smokers. The Alpha-Tocopherol, Beta Carotene Cancer Prevention Study Group. N Engl J Med.

[12] Martínez ME, Jacobs ET, Baron JA, Marshall JR, Byers T (2012). Dietary supplements and cancer prevention: balancing potential benefits against proven harms. J Natl Cancer Inst.

[13] Mabuchi H, Nohara A, Kobayashi J, Kawashiri MA, Katsuda S, Inazu A, Hokuriku Lipid Research Group (2007). Effects of CoQ10 supplementation on plasma lipoprotein lipid, CoQ10 and liver and muscle enzyme levels in hypercholesterolemic patients treated with atorvastatin: a randomized double-blind study. Atherosclerosis.

[14] Seely D, Kanji S, Yazdi F, Tetzlaff J, Singh K, Tsertsvadze A (2012). Dietary supplements in adults taking cardiovascular drugs.

[15] Baygi F, Sotoudeh G, Qorbani M, Sadrzadeh-Yeganeh H, Rahimi A, Koohdani F (2013). Predictors of dietary supplement use among female health workers in Tehran. J Diabetes Metab Disord.

[16] Saeedi P, Mohd Nasir MT, Hazizi AS, Vafa MR, Rahimi Foroushani A (2013). Nutritional supplement use among fitness club participants in Tehran, Iran. Appetite.

[17] Darvishi L, Askari G, Hariri M, Bahreynian M, Ghiasvand R, Ehsani S (2013). The use of nutritional supplements among male collegiate athletes. Int J Prev Med.

[18] Yong MB (1990). Vitamin use and beliefs among students at a Malaysian university. J R Soc Health.

[19] Spencer EH, Bendich A, Frank E (2006). Vitamin and mineral supplement use among US medical students: a longitudinal study. J Am Diet Assoc.

[20] Suleiman AA, Alboqai OK, Yasein N, Al-Essa MK, El Masri K (2008). Prevalence of vitamin-mineral supplement use among Jordan University students. Saudi Med J.

[21] Ock S-M, Hwang S-S, Lee J-S, Song C-H, Ock C-M (2010). Dietary supplement use by South Korean adults: Data from the national complementary and alternative medicine use survey (NCAMUS) in 2006. Nutr Res Pract.

[22] Foote JA, Murphy SP, Wilkens LR, Hankin JH, Henderson BE, Kolonel LN (2003). Factors associated with dietary supplement use among healthy adults of five ethnicities: the multiethnic cohort study. Am J Epidemiol.

[23] Radimer K, Bindewald B, Hughes J, Ervin B, Swanson C, Picciano MF (2004). Dietary supplement use by US adults: data from the National Health and Nutrition Examination Survey, 1999-2000. Am J Epidemiol.

[24] Marques-Vidal P (2004). Vitamin supplement usage and nutritional knowledge in a sample of Portuguese health science students. Nutr Res.

[25] Pérusse-Lachance E, Tremblay A, Drapeau V (2010). Lifestyle factors and other health measures in a Canadian university community. Appl Physiol Nutr Metab.

[26] von Bothmer MI, Fridlund B (2005). Gender differences in health habits and in motivation for a healthy lifestyle among Swedish university students. Nurs Health Sci.

[27] Tirodimos I, Georgouvia I, Savvala TN, Karanika E, Noukari D (2009). Healthy lifestyle habits among Greek university students: differences by sex and faculty of study. East Mediterr Health J.

[28] Ranelli PL, Dickerson RN, White KG (1993). Use of vitamin and mineral supplements by pharmacy students. Am J Hosp Pharm.

[29] Gunther S, Patterson RE, Kristal AR, Stratton KL, White E (2004). Demographic and health-related correlates of herbal and specialty supplement use. J Am Diet Assoc.

[30] Kirk SF, Cade JE, Barrett JH, Conner M (1999). Diet and lifestyle characteristics associated with dietary supplement use in women. Public Health Nutr.

[31] Frank E, Bendich A, Denniston M (2000). Use of vitamin-mineral supplements by female physicians in the United States. Am J Clin Nutr.

[32] Ervin RB, Wright JD, Kennedy-Stephenson J (1999). Use of dietary supplements in the United States, 1988-1994. Vital Health Stat.

[33] Lyle BJ, Mares-Perlman JA, Klein BE, Klein R, Greger JL (1998). Supplement users differ from nonusers in demographic, lifestyle, dietary and health characteristics. J Nutr.

[34] Dickinson A, Shao A, Boyon N, Franco JC (2011). Use of dietary supplements by cardiologists, dermatologists and orthopedists: report of a survey. Nutr J.

[35] Robson PJ, Siou GL, Ullman R, Bryant HE (2008). Sociodemographic, health and lifestyle characteristics reported by discrete groups of adult dietary supplement users in Alberta, Canada: findings from The Tomorrow Project. Public Health Nutr.

[36] Ashar BH, Rice TN, Sisson SD (2008). Medical residents’ knowledge of dietary supplements. J.

[37] Kato I, Nomura AM, Stemmermann GN, Chyou PH (1992). Vitamin supplement use and its correlates among elderly Japanese men residing on Oahu, HI. Public Health Rep.

[38] Fennell MA (2004). Determinants of supplement usage. Prev Med.

